# Transgenic banana plants expressing *Xanthomonas* wilt resistance genes revealed a stable non-target bacterial colonization structure

**DOI:** 10.1038/srep18078

**Published:** 2015-12-10

**Authors:** Jean Nimusiima, Martina Köberl, John Baptist Tumuhairwe, Jerome Kubiriba, Charles Staver, Gabriele Berg

**Affiliations:** 1 National Agricultural Research Organisation, National Agricultural Research Laboratories, Kampala, Uganda; 2 Makerere University, College of Agricultural and Environmental Sciences, Department of Agricultural Production, Kampala, Uganda; 3 Graz University of Technology, Institute of Environmental Biotechnology, Austria; 4 Bioversity International, Montpellier, France

## Abstract

Africa is among the continents where the battle over genetically modified crops is currently being played out. The impact of GM in Africa could potentially be very positive. In Uganda, researchers have developed transgenic banana lines resistant to banana *Xanthomonas* wilt. The transgenic lines expressing *hrap* and *pflp* can provide a timely solution to the pandemic. However, the impact of the transgenes expression on non-target microorganisms has not yet been investigated. To study this effect, transgenic and control lines were grown under field conditions and their associated microbiome was investigated by 16S rRNA gene profiling combining amplicon sequencing and molecular fingerprinting. Three years after sucker planting, no statistically significant differences between transgenic lines and their non-modified predecessors were detected for their associated bacterial communities. The overall gammaproteobacterial rhizosphere microbiome was highly dominated by *Xanthomonadales*, while *Pseudomonadales* and *Enterobacteriales* were accumulated in the pseudostem. Shannon indices revealed much higher diversity in the rhizosphere than in the pseudostem endosphere. However, the expression of the transgenes did not result in changes in the diversity of *Gammaproteobacteria*, the closest relatives of the target pathogen. In this field experiment, the expression of the resistance genes appears to have no consequences for non-target rhizobacteria and endophytes.

Banana *Xanthomonas* wilt (BXW) triggered by the plant pathogen known as *Xanthomonas campestris* pathovar *musacearum* is a highly devastating disease in banana production, ranked first in the Great Lakes region of East and Central Africa^1^,^2^,^3^,^4^. The economic impact of the banana wilt has been disastrous, because it affects almost all commonly grown banana cultivars, leading to yield collapse as it continues to spread. Currently, there are no commercial pesticides, biological control agents or resistant banana cultivars available to bring the wilting disease under control, although rigorous cultural practices have been shown to minimize disease damage^5^,^6^. Due to carryover of soil-borne inoculum, infested fields cannot be replanted with bananas for at least half a year^2^. To address this problem, Tripathi *et al.*^7^ and Namukwaya *et al.*^8^ from the International Institute of Tropical Agriculture (IITA) and the National Agricultural Research Organisation (NARO) in Uganda have developed transgenic banana lines with resistance to BXW mediated by the constitutive expression of the resistance genes *hrap* (hypersensitive response assisting protein) and *pflp* (plant ferredoxin-like protein), both originating from sweet pepper *Capsicum annuum*. These genetically modified banana lines have already proven their enhanced resistance against *X. campestris* pv. *musacearum* under greenhouse^7^,^8^ and field conditions^6^. Both transgenes are associated with the harpin-elicited hypersensitive response (HR) in plants challenged with Gram-negative pathogens. Hrap intensifies the HR activation by dissociating harpin multimers into dimers and monomers which triggers a stronger hypersensitive cell death (HCD) necrosis leading to a systemic acquired resistance (SAR) of the plant^9^,^10^. Overexpression of Pflp leads to increased production of reactive oxygen species (ROS) in harpin-activated cells and consequently to HCD and SAR^11^. The lack of natural resistance to BXW in any banana cultivar and the difficulties in conventional breeding with this highly sterile crop favor an effective transgenic approach. However, nothing is known about non-target effects, especially on *Gammaproteobacteria*, the closest relatives of the target pathogen.

Globally, transgenic or genetically modified (GM) crops are considered regulated products subject to regulatory oversight during testing and environmental release. The main concern about GM crops centers on the lack of studies on the side effects, in terms of adverse impacts on the environment and human health, although substantial research was performed in Europe^12^,^13^,^14^,^15^. The controversies surrounding GM crops highlight the need to establish regulatory frameworks and data of these technologies in Africa^16^,^17^,^18^. Despite clear benefits to countries and farmers who grow GM crops, there is concern about suspected potential risks associated with GMOs^19^,^20^. To move beyond the 30 years debates on existing GM crops, where they are often discussed in “black and white”, we need a bigger picture which is “nuanced, equivocal and undeniably messy”, a data-driven debate as well as risk assessment studies for GM crops also for Africa^17^.

In this respect, we analyzed the plant-associated microbiome of transgenic banana lines expressing sweet pepper *hrap* and *pflp* genes in comparison to their not genetically modified predecessors grown under natural soil conditions in a confined field trial in Uganda. The experiment was a randomized complete block design (RCBD) with two different breeding lines of the East African Sukari Ndizi (AAB genome). Each breeding line expressing each of the transgenes was replicated four times and had four non-transgenic control plants (Fig. S1). We monitored the effect of the transgenes on the composition and diversity of the banana-associated microbiome in the rhizosphere and the pseudostem endosphere, with special focus on the gammaproteobacterial community which comprises the causal agent of BXW.

## Results

### Molecular fingerprinting of total bacterial communities

**Figure 1:**
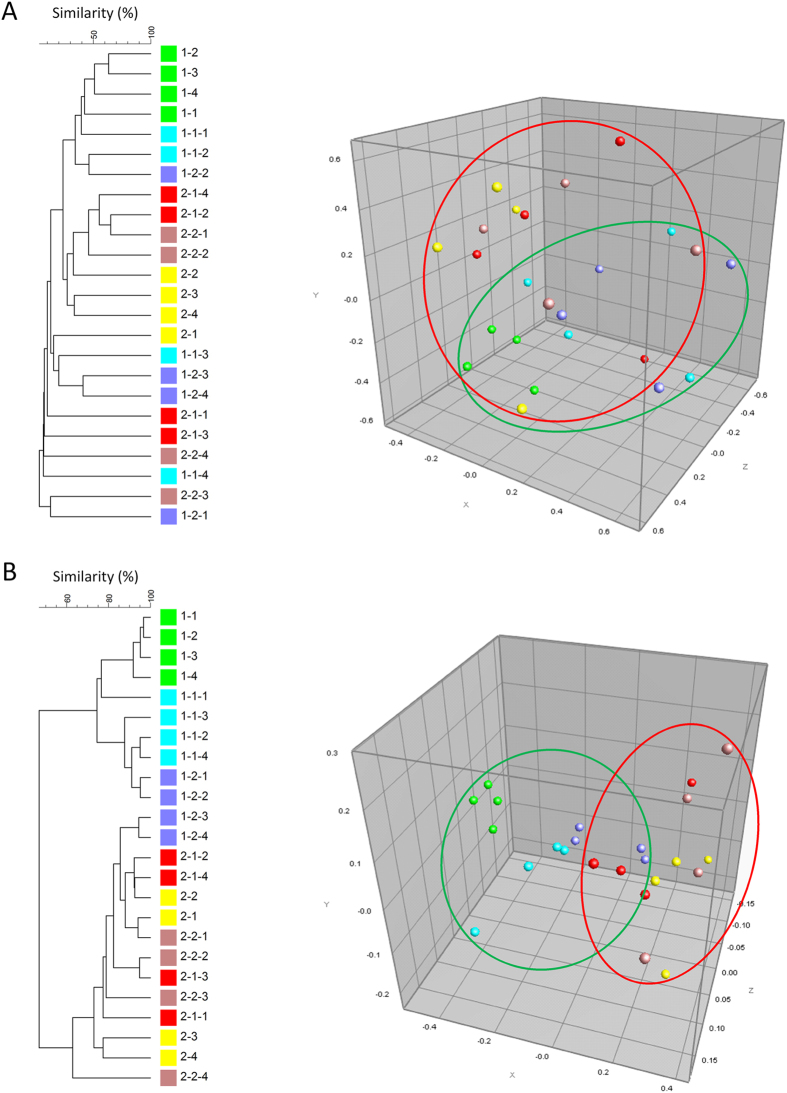
Comparative analyses of PCR-SSCP profiles of the total bacterial communities in rhizosphere soil (A) and pseudostem (B) of transgenic and non-transgenic banana plants. Left: Unweighted pair group method with arithmetic mean (UPGMA) trees. The dendrograms were generated with GelCompar II using Pearson correlation. Samples were encoded using abbreviations indicating (1) breeding line (1, 2), (2) genetic modification, if any (1 = *hrap*, 2 = *pflp*), and (3) independent replicate sample (1–4). Right: Multidimensional scaling (MDS) ordination plots based on Pearson similarity matrices. Colors indicate genetic modification and correspond to squares in the tree, and samples of the two investigated breeding lines are grouped together.

Molecular fingerprinting of the total bacterial community associated with genetic-modified banana plants and their non-modified predecessors using SSCP analysis showed significant differences in their endophytic colonization of the pseudostem between the two investigated breeding lines (p < 0.001, permutation test) (Fig. 1). High variability was found in the rhizospheric soil communities within the breeding lines. No significant effect from genetic modification on the banana-associated bacterial microbiome was found in either the rhizosphere or the endosphere. The rhizosphere soil exhibited a highly diverse bacterial community composition, while the pseudostem endosphere was characterized by a much lower abundance of species. Interestingly, different species of the genus *Methylobacterium* (closest database matches *M. mesophilicum*, *M. phyllosphaerae*, and *M. adhaesivum*) were found as dominant endophytic banana colonizers in all investigated pseudostem samples. Furthermore, *Bacillus* (closest database match *B. flexus*, 100% similarity to GenBank accession number NR_024691) and *Paenibacillus* (closest database match *P. barcinonensis*, 100% similarity to GenBank accession number NR_042272) were identified as dominant banana endophytes.

### Amplicon sequencing-based 16S rRNA gene profiling of the gammaproteobacterial community

The 796,445 quality sequences with a read length ≥200 nucleotides (between 1,594 and 43,012 quality reads per sample), generated through deep sequencing-based analysis, provided detailed insights into the gammaproteobacterial community composition and diversity. Rarefaction analyses of the normalized *Gammaproteobacteria*-specific sequencing data at three different cut-off levels (3%, 5%, and 10% genetic dissimilarity), corresponding to the taxonomic levels of species, genera and families (Fig. S2) showed higher numbers of operational taxonomic units (OTUs) in the rhizosphere than in the endophytic pseudostem tissue. Comparisons of observed OTUs with their estimated richness by the Chao1 index revealed relatively high coverages for the individual samples between 58.4 and 100% (S: 58.4–77.1%; P: 72.8–100%) at order level (Table S1). The sequencing efforts at genus and species level reached 42.5–84.7% (S: 42.5–60.9%; P: 45.1–84.7%) and 30.4–66.1 (S: 34.3–56.7; P: 30.4–66.1%), respectively. The rhizosphere soil exhibited a significantly higher diversity within the gammaproteobacterial community in comparison to the pseudostem of the banana plants (p < 0.001, *t*-test). Shannon diversity indices (H’) for the rhizosphere samples ranged from 5.12 to 6.86 at a dissimilarity level of 3%, while values for endosphere samples were in the range of 1.53 to 3.14 (Table S1). The expression of the transgenes *hrap* and *pflp* did not result in changes in gammaproteobacterial diversity (S: p = 0.525; P: p = 0.979, Tukey *post hoc* test).

**Figure 2:**
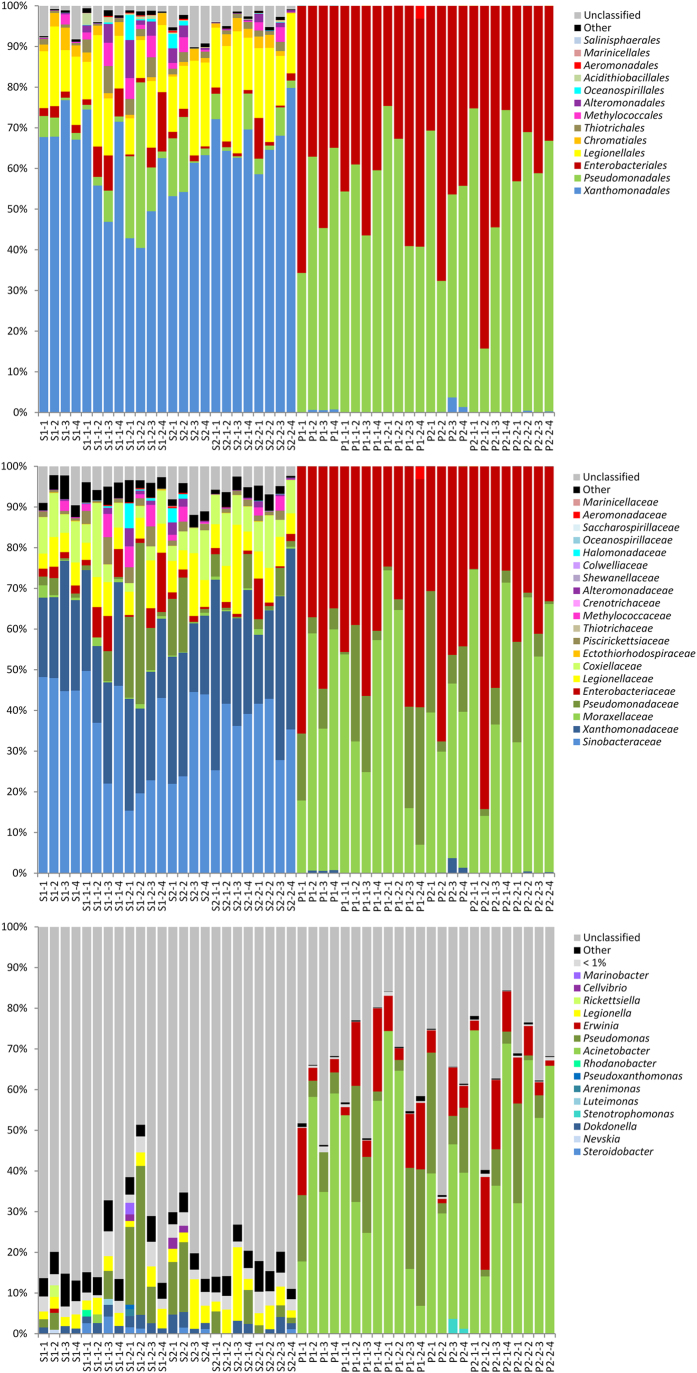
Taxonomic composition of the gammaproteobacterial communities inhabiting rhizosphere soil (S) and pseudostem (P) of banana plants with and without genetic modifications. Sequences obtained by Illumina MiSeq sequencing were classified at order, familiy and genus level. From each genetic modification, four independent replicate samples were investigated in comparison to non-modified control plants. Sample abbreviations indicate: (1) microenvironment (S = rhizosphere soil, P = pseudostem), (2) breeding line (1, 2), (3) genetic modification, if any (1 = *hrap*, 2 = *pflp*), and (4) independent replicate sample (1–4).

While all quality sequences from the pseudostem could be assigned at least to a gammaproteobacterial family, in the rhizosphere soil, on average, 3.4% per sample could not be taxonomically assigned accurately below the class level, and 5.4% not below the order level (Fig. 2). At genus level, the pseudostem reads which could be unambiguously affiliated to a gammaproteobacterial genus were much greater than for rhizospheric reads, 64.2% versus 21.1%. Highest abundances in the rhizosphere soil were found for *Xanthomonadales* (average 62.3% per sample), *Legionellales* (16.3%), *Pseudomonadales* (6.8%), and *Enterobacteriales* (3.2%) 16S rRNA gene sequences. The overall accumulation of *Pseudomonadales* (54.8%) and *Enterobacteriales* (44.7%) in the pseudostem endosphere of the banana plants was notable.

At lower taxonomic levels, *Xanthomonadales* could be assigned to *Sinobacteraceae* (genera *Steroidobacter*, and *Nevskia*), and *Xanthomonadaceae* (genera *Dokdonella*, *Stenotrophomonas*, *Luteimonas*, *Arenimonas*, *Pseudoxanthomonas*, and *Rhodanobacter*). *Pseudomonadales* reads could be classified in *Moraxellaceae* (*Acinetobacter*) and *Pseudomonadaceae* (*Pseudomonas*). The enterobacterial fraction was dominated by *Erwinia*, and the order *Legionellales* could be divided into the families *Legionellaceae* (*Legionella*) and *Coxiellaceae* (*Rickettsiella*). Further genera identified for taxonomic groups with a relative abundance over 1% in any sample belonged to the *Alteromonadales* (*Cellvibrio*, and *Marinobacter*), exclusively found in some rhizosphere samples.

**Figure 3:**
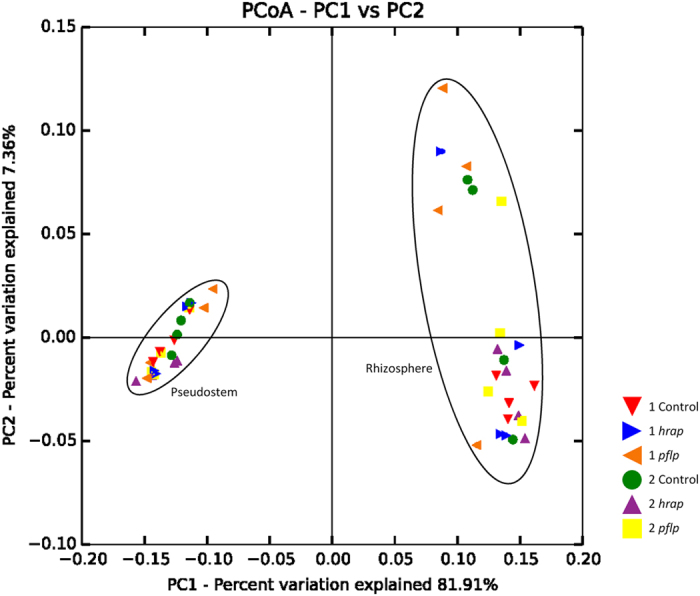
Principal coordinate analysis (PCoA) plot of the gammaproteobacterial microbiome inhabiting rhizosphere soil and pseudostem of two different banana breeding lines (1 and 2) expressing different transgenes (*hrap* and *pflp*). PCoA biplots are based on weighted UniFrac distances of gammaproteobacterial 16S rRNA gene amplicon sequencing data jackknife-supported by ten replicates. Statistical comparisons based on the underlying distance matrices are shown in the supplementary information (Tables S2).

Principal coordinate analysis based on weighted UniFrac distances visualized a clear separation of the two investigated microenvironments, rhizosphere and endosphere, resulting from the remarkable differences in their hosted *Gammaproteobacteria* communities (Fig. 3). The statistical significance was additionally confirmed by adonis test (p = 0.001). In contrast to the highly similar gammaproteobacterial community profiles found in pseudostem samples, the rhizosphere samples showed a much broader scattering. Within microenvironments, no statistically significant differences could be observed between the two individual breeding lines (S: p = 0.249; P: p = 0.643, adonis test) nor between plants with genetic modifications and their non-modified predecessors (p > 0.05, adonis test; Table S2).

## Discussion

In our risk assessment study for transgenic banana lines resistant to banana *Xanthomonas* wilt (BXW), we investigated two microenvironments and found statistically significant differences for the composition and diversity of rhizosphere and endosphere bacterial communities. This shows that the applied sampling design and the methods were appropriate to detect statistical differences. The importance of rare taxa for bacterial diversity, shown recently in the rhizosphere of *Bt*- and conventional maize varieties^15^, was established. Three years after sucker planting, we found no differences between transgenic lines and their non-modified predecessors, indicating that in our field experiment the insertion of the BXW resistance genes in the banana genome appears to have no consequences for non-target rhizobacteria and endophytes of healthy banana plants. Longer-term studies are needed to track further changes.

The rhizosphere microbiome is mainly a result of microbe attraction by root secretions and other rhizodeposits released by the plant. These chemical stimulants are highly controlled by the plant genotype^21^,^22^,^23^. The presence and expression of *hrap* and *pflp* genes in transgenic banana plants exhibited no impact in this study on composition and diversity within the bacterial rhizosphere microbiome. This suggests that inserting these genes in banana lines has not significantly affected chemical functioning of root exudates or that rhizobacteria compensate these changes. *Xanthomonadales* was the predominant gammaproteobacterial order in the rhizosphere of transgenic and non-transgenic banana plants. In contrast in the rhizosphere of dessert bananas investigated in Central America, where the BXW disease is not present^2^,^3^, *Pseudomonadales* and *Legionellales* were observed as the most dominant gammaproteobacterial orders^24^. Several members of *Xanthomonadales* are known as phytopathogens that cause a variety of serious diseases in a number of crops, including banana which is threatened by BXW^6^,^25^. In most rhizosphere samples for both the transgenic and non-transgenic lines, the *Xanthomonadales* family *Sinobacteraceae* dominated over *Xanthomonadaceae*; the genus *Xanthomonas* was not found in any sample. Instead, *Dokdonella* was the most identified genus within the *Xanthomonadaceae* family, and *Steroidobacter* within the *Sinobacteraceae*, which are both common soil bacteria and not associated with plant pathogenicity. However, only a relatively small proportion of sequences could be classified down to the genus level, and samples originated only from healthy banana plants without disease symptoms. Evidence that the community composition of the rhizosphere microbiome was not influenced by constitutive expression of transgenic BXW resistance genes indicates that the useful key contributions of banana root exudates to the rhizosphere ecology were not affected.

In the inner plant tissue, microorganisms are protected against the competitive and sometimes hostile rhizosphere environment. In turn, endophytes have often close and advantageous interactions with their host plants^26^. The endosphere of the succulent banana pseudostem has been found to be an extraordinary microenvironment due to its generally dense bacterial colonization and the presence of a remarkably high number (9.4%) and broad spectrum of antagonistic strains^27^. The dominant genera among the banana endophytes, *Methylobacterium*, *Bacillus*, and *Paenibacillus*, revealed through molecular fingerprinting have all been previously detected as endophytes of a variety of plants, primarily associated with beneficial plant-microbe interactions. A broad diversity of *Methylobacterium* spp. was, for instance, observed for the endosphere of citrus plants, where they were described as main players in interactions with the phytopathogen *Xylella fastidiosa*, also belonging to the *Xanthomonadaceae* family^28^,^29^. Endophytic *Bacillus* and *Paenibacillus* isolates of medicinal plants were identified as being amongst the most efficient broad-spectrum antagonists against soil-borne plant diseases of Egypt^30^. The apparent lack of interference of the transgenic banana lines expressing *hrap* and *pflp* genes on endophytic interactions is an important achievement which should be taken into account broadly in breeding programs. Just as with the rhizosphere, the endosphere community remained stable, irrespective of the foreign genes inserted into the banana genome. While the gammaproteobacterial rhizosphere colonization was highly dominated by *Xanthomonadales*, the banana endosphere was almost exclusively inhabited by *Pseudomonadales* and *Enterobacteriales*. The classifiable pseudostem endophytes were assigned to the genera *Acinetobacter*, *Pseudomonas*, and *Erwinia*. All of them are well-known plant colonizers. However, while *Acinetobacter* and *Pseudomonas* are often accountable for disease-suppressive antagonism, plant growth promotion and stress reduction^31^,^32^, *Erwinia* has so far mainly been recognized as a phytopathogen, causing, for instance, the devastating fireblight disease in *Rosaceae* plants^33^,^34^ and pseudostem wet rot in plantain^35^. The high presence in the pseudostem of fecal enterobacteria could be explained by the manure applications used in the experiment twice a year. Manure applications, widely used in Uganda’s banana production, affect the native soil and plant-associated microbiome, and a masking of potential transgene effects cannot be completely excluded under these circumstances. The endophytic gammaproteobacterial colonization patterns are well in accordance with those found for the Gros Michel banana investigated in Central America^24^ and the East African Highland banana of Uganda^27^. However, the difference in the rhizosphere, which in this field trial was so highly inhabited by *Xanthomonadales*, and the rigorous selection process of enterics and pseudomonads was even more notable in the present study compared to previous studies.

It is crucial that the release of GM crops does not bring new risks with irretrievable consequences for environmental and human health. In our short-term study, we found no detectable impact on the inhabiting bacterial communities resulting from any genetic modification, by the expression either of the *hrap* or of the *pflp* transgene. Additional studies should address the microbiome stability over a longer time frame, confirm the stability in other soil types and under different management practices, and investigate the effects in the presence of the disease.

This encouraging result, however, also serves to remind us that the agricultural challenges facing the developing world are very broad and diverse^16^. Investment in soil management to improve soil fertility and resilience through alternative approaches already established in Africa like agroforestry, intercropping and crop-livestock integration need to be considered, including their effects on the soil and plant microbiome. Moreover, in the push for greater productivity often achieved through specialization, we have the opportunity to avoid the loss in diversity of human gut microbiota found in the developed world compared to the higher diversity found in African people^36^,^37^, acknowledged to be under the influence of diets and lifestyles.

## Methods

### Experimental design and sampling

The study was carried out on an on-going confined field trial of transgenic bananas at the National Agricultural Research Laboratories (NARL) located about 13 km north of Kampala at an altitude of 1,190 m above sea level. Average annual rainfall is 1,250 mm distributed bimodally, and the annual temperature is 27.3/15.3 °C (mean maximum/mean minimum) with only 1–2 degrees difference between coolest and warmest months. The clay soil where the transgenic banana trial was established was slightly acidic (pH 5.2) with low nutrient content. The cultivar under study was the East African Sukari Ndizi (AAB genome) with two different breeding lines originating from tissue culture, each expressing vector-inserted *hrap* (hypersensitive response assisting protein) and *pflp* (plant ferredoxin-like protein) genes^7^,^8^. Tissue culture plants were planted at a spacing of (3 × 3) m. The experiment was a randomized complete block design (RCBD) organized in four block repetitions with six genotypically different plants per block (Fig. S1). Each breeding line expressing each of the genes was replicated four times and had four non-transgenic control plants. Guard row plants were planted around the experimental plot. Cow dung manure at a rate of 10,000 kg ha^−1^ was applied at planting. Both cow manure and mulch were applied subsequently twice a year at the same rates as at planting. Banana plant and field management, de-trashing, de-suckering, de-budding and weeding, were done monthly. The plantation was three years old at sampling time. From each plant, samples were collected from both the rhizosphere and the pseudostem endosphere and stored under cooled conditions until workup in the laboratory.

### Metagenomic DNA isolation

To isolate total community DNA, 2 g of each rhizosphere soil sample and 15 ml of sterile 0.85% NaCl were mixed for 10 sec on the vortex. For the isolation from the banana endosphere, 5 g of pseudostem were washed with sterile distilled water, transferred to Whirl-Pak bags (Nasco, Fort Atkinson, WI, USA), and after 10 ml 0.85% NaCl were added, homogenized using mortar and pestle. From the liquid parts, 4 ml were centrifuged at high speed (16,000 × g, 4 °C) for 20 min and resulting pellets were stored at −70 °C. Total community DNA was extracted using the FastDNA SPIN Kit for Soil (MP Biomedicals, Solon, OH, USA) according to the manufacturer’s protocol. Metagenomic DNA samples were encoded using abbreviations indicating: (1) microenvironment (S = rhizosphere soil, P = pseudostem), (2) breeding line (1, 2), (3) genetic modification, if any (1 = expressing *hrap* gene [hypersensitive response assisting protein], 2 = expressing *pflp* gene [plant ferredoxin-like protein]), and (4) independent replicate sample (1–4).

### Fingerprinting of the total bacterial community by single-stranded conformational polymorphism analysis of the 16S rRNA genes (PCR-SSCP)

Fingerprinting by SSCP analysis was carried out as described by Schwieger & Tebbe^38^. Bacterial 16S rRNA gene sequences were PCR-amplified using the eubacterial primer pair Unibac-II-515f and Unibac-II-927r^P^. Separation and analysis were performed according to Köberl *et al.*^39^. Comparisons of generated bacterial community profiles were performed using GelCompar II 5.1 (Applied Maths, Kortrijk, Belgium). Cluster analyses were performed with the following settings: dendrogram type: unweighted pair group method with arithmetic mean (UPGMA); similarity coefficient: curve based: Pearson correlation; position tolerances: optimisation: 0.2%, position tolerance: 1%. Multidimensional scaling (MDS) ordination plots were constructed based on the Pearson similarity matrices. These matrices were additionally subjected to significance tests of pair-wise similarities by applying permutation analyses (p ≤ 0.01) using the permtest package of R statistics 3.2.0 (The R Foundation for Statistical Computing, Vienna, Austria) with 10^5^ random permutations of sample elements^40^,^41^. Excised and re-amplified DNA fragments were sequenced at LGC Genomics (Berlin, Germany).

### Gammaproteobacterial 16S rRNA gene profiling by Illumina MiSeq Sequencing

In a deep-sequencing approach, we focused on the colonization by *Gammaproteobacteria*. The hypervariable V4 region of the 16S rRNA gene was amplified in a nested PCR approach with the *Gammaproteobacteria* specific primer pair Gamma395f/Gamma871r^42^ and the universal primer pair 515F/806R^43^ according to Köberl *et al.*^24^. PCR products of three independent reactions were pooled in equal volumes and purified by employing the Wizard SV Gel and PCR Clean-Up System (Promega, Madison, WI, USA). Amplicon libraries were generated and sequenced by a paired-end approach using the Illumina MiSeq platform (LGC Genomics, Berlin, Germany). The nucleotide sequences are available in the European Nucleotide Archive (www.ebi.ac.uk/ena) under the BioProject accession number PRJEB9422.

Data analysis was performed by employing the software package QIIME 1.7 and 1.8^44^. Joined paired-end reads with more than three consecutive low quality base calls (Phred quality score ≤20) were truncated at the position where their quality began to drop, and only reads with >75% consecutive high quality base calls, without any ambiguous characters, and longer than 200 nucleotides in length were retained for further analyses. All quality sequences were adjusted in the same orientation and clustered into operational taxonomic units (OTUs) with uclust^45^, using 3%, 5%, and 10% dissimilarity thresholds. From each OTU the most abundant sequence was selected as the representative one, and the taxonomy of the representative set was assigned with the uclust-based consensus taxonomy assigner using an 80% confidence threshold. The representative sequence set was aligned with PyNAST^46^. Chimera check was performed with ChimeraSlayer and potentially chimeric sequences were discarded. OTU tables at the different dissimilarity levels were constructed, and OTUs not assigned to the class of *Gammaproteobacteria* as well as singletons were removed from the dataset. For alpha and beta diversity analyses, OTU tables were rarefied at 1,590 reads. Diversity indices Shannon^47^, Chao1^48^ and observed species were determined based on the normalized clustering data. Significant differences were calculated with PASW Statistics 18 (SPSS Inc., Chicago, IL, USA) using the independent samples *t*-test and the Tukey *post hoc* test. Beta diversity was analyzed based on weighted UniFrac distances^49^ and ten jackknife replicates of the total rarefied datasets. Statistical analyses were performed using the adonis test (p ≤ 0.05) with 999 permutations.

## Additional Information

**How to cite this article**: Nimusiima, J. *et al.* Transgenic banana plants expressing *Xanthomonas* wilt resistance genes revealed a stable non-target bacterial colonization structure. *Sci. Rep.* **5**, 18078; doi: 10.1038/srep18078 (2015).

## Supplementary Material

Supplementary Information
