# Supplementary material for: Transgenic banana plants expressing Xanthomonas wilt resistance genes revealed a stable non-target bacterial colonization structure
Source: Sci Rep. 2015 Dec 10;5:18078. doi: 10.1038/srep18078 (PMC4674801; doi:10.1038/srep18078)
Supplement: Supplementary Information [file srep18078-s1.pdf]

Transgenic banana plants expressing *Xanthomonas* wilt resistance genes revealed a stable non-target bacterial colonization structure

Jean Nimusiima[1,2,+], Martina Köberl[3,+], John Baptist Tumuhairwe[2], Jerome Kubiriba[1], Charles Staver[4], Gabriele Berg[3]

[1]*National Agricultural Research Organisation, National Agricultural Research Laboratories, Kampala, Uganda*

[2]*Makerere University, College of Agricultural and Environmental Sciences, Department of Agricultural Production, Kampala, Uganda*

[3]*Graz University of Technology, Institute of Environmental Biotechnology, Austria*

[4]*Bioversity International, Montpellier, France*

[+]these authors contributed equally to this work

Table S1. Richness estimates and diversity indices for gammaproteobacterial 16S rRNA gene amplicon libraries at 3%, 5%, and 10% genetic dissimilarity.

Sample[a]	Quality reads[b]	Clusters[c] (OTUs)			Chao1 (OTUs)			Coverage (%)			Shannon (H')		
		3%	5%	10%	3%	5%	10%	3%	5%	10%	3%	5%	10%
S1-1	1,590	293	200	85	572	362	115	51.2	55.2	73.6	6.16	5.17	3.74
S1-2	1,590	278	201	85	580	373	125	47.9	53.8	67.9	5.74	4.88	3.56
S1-3	1,590	246	166	72	540	342	107	45.6	48.7	66.8	5.39	4.55	3.13
S1-4	1,590	287	201	82	540	359	112	53.1	56.1	72.9	5.98	4.95	3.55
S1-1-1	1,590	210	144	68	441	268	88	47.6	53.7	77.1	5.53	4.90	3.58
S1-1-2	1,590	237	168	71	488	313	98	48.6	53.8	72.0	5.89	5.16	3.77
S1-1-3	1,590	370	264	107	816	581	166	45.3	45.5	64.1	6.79	6.11	4.86
S1-1-4	1,590	254	163	70	555	322	100	45.8	50.8	69.8	5.50	4.57	3.26
S1-2-1	1,590	272	194	87	663	384	124	41.0	50.5	69.5	5.80	5.29	4.36
S1-2-2	1,590	299	204	82	703	413	121	42.5	49.4	67.8	5.70	4.93	3.61
S1-2-3	1,590	347	258	95	860	522	139	40.4	49.3	68.0	6.61	6.02	4.58
S1-2-4	1,590	192	137	67	346	226	92	55.4	60.9	72.6	5.12	4.49	3.27
S2-1	1,590	313	213	92	606	384	145	51.6	55.4	63.3	6.45	5.61	4.37
S2-2	1,590	339	239	86	831	535	148	40.8	44.6	58.4	6.61	5.82	4.25
S2-3	1,590	197	139	57	373	236	75	52.8	59.1	76.5	5.40	4.57	3.35
S2-4	1,590	302	210	87	589	397	121	51.2	52.9	71.6	6.43	5.54	4.01
S2-1-1	1,590	231	164	65	408	290	89	56.7	56.6	73.4	5.17	4.37	3.19
S2-1-2	1,590	323	224	84	644	395	115	50.1	56.8	73.0	6.33	5.36	3.97
S2-1-3	1,590	230	159	63	481	308	92	47.8	51.4	68.6	5.38	4.54	3.26
S2-1-4	1,590	284	192	82	555	348	113	51.2	55.2	72.3	6.12	5.10	3.70
S2-2-1	1,590	270	198	84	479	346	124	56.4	57.1	67.7	6.25	5.46	3.88
S2-2-2	1,590	334	236	87	728	471	141	45.9	50.2	61.6	6.35	5.38	3.90
S2-2-3	1,590	408	304	106	1191	714	152	34.3	42.5	69.8	6.86	6.12	4.39
S2-2-4	1,590	360	259	103	806	495	148	44.7	52.4	69.1	6.07	5.10	3.73
P1-1	1,590	87	27	6	178	43	8	49.0	62.0	72.8	2.97	2.03	1.31
P1-2	1,590	63	26	8	139	48	8	45.2	54.4	92.7	2.58	1.83	1.24
P1-3	1,590	59	25	9	132	35	11	44.9	70.4	80.6	2.42	1.98	1.40
P1-4	1,590	47	21	7	152	31	8	31.2	66.9	89.2	2.00	1.74	1.27
P1-1-1	1,590	41	14	5	97	18	6	42.4	80.0	90.9	1.89	1.40	1.05
P1-1-2	1,590	38	19	3	85	25	3	44.9	77.0	100	2.48	2.16	1.57
P1-1-3	1,590	56	22	4	153	33	4	36.9	68.1	94.7	2.44	1.96	1.44
P1-1-4	1,590	48	16	4	79	29	4	60.6	55.0	97.3	2.27	1.70	1.10
P1-2-1	1,590	47	13	4	83	15	4	56.8	84.7	97.6	1.80	1.26	0.91
P1-2-2	1,590	53	15	3	101	19	3	52.7	76.2	97.0	2.15	1.45	1.08
P1-2-3	1,590	81	24	4	146	46	4	55.5	50.6	97.7	2.98	2.09	1.39
P1-2-4	1,590	99	28	5	204	42	7	48.6	66.5	77.9	3.14	1.96	1.26
P2-1	1,590	64	26	5	171	49	7	37.4	52.4	73.5	2.45	2.06	1.59
P2-2	1,590	51	13	7	101	19	9	50.7	68.4	82.4	1.70	1.48	1.07
P2-3	1,590	65	24	9	211	44	9	31.0	55.1	98.9	2.91	2.31	1.53
P2-4	1,590	57	27	9	127	47	12	45.1	57.4	80.7	2.61	2.08	1.59
P2-1-1	1,590	44	14	4	69	17	4	64.2	80.1	97.2	2.20	1.33	0.84
P2-1-2	1,590	83	17	4	126	23	4	66.1	75.5	97.4	2.75	1.41	0.71
P2-1-3	1,590	69	22	5	128	35	5	54.0	62.6	98.0	2.82	1.94	1.30
P2-1-4	1,590	59	22	5	125	34	5	47.2	63.6	93.2	2.10	1.63	1.01
P2-2-1	1,590	90	27	6	196	60	8	46.1	45.1	73.9	3.05	2.17	1.57
P2-2-2	1,590	53	24	8	131	40	8	40.7	59.3	90.9	2.09	1.80	1.02

P2-2-3	1,590	82	26	4	271	54	4	30.4	46.9	98.8	2.53	1.96	1.27
P2-2-4	1,590	36	16	6	99	23	7	36.9	71.3	94.8	1.53	1.38	1.00

[a]Sample abbreviations indicate: (1) microenvironment (S = rhizosphere soil, P = pseudostem), (2) breeding line (1, 2), (3) genetic modification, if any (1 = *hrap*, 2 = *pflp*), and (4) independent replicate sample (1-4).
[b]quality reads were normalized to the same number of sequences.
[c]rarefaction curves are depicted in Fig. S2.

Table S2. Statistical comparisons of the gammaproteobacterial communities inhabiting two banana breeding lines both bred with and without *hrap* and *pflp* gene insertion.

	**Breeding line 1**		**Breeding line 2**	
	**Rhizosphere**	**Pseudostem**	**Rhizosphere**	**Pseudostem**
**Control – *hrap***	0.435	0.661	0.263	0.066
**Control – *pflp***	0.139	0.655	0.590	0.271
hrap – pflp	0.395	0.939	0.527	0.422

p values for pairwise comparisons between different genotypes ascertained by adonis test based on jackknifed weighted UniFrac distances.

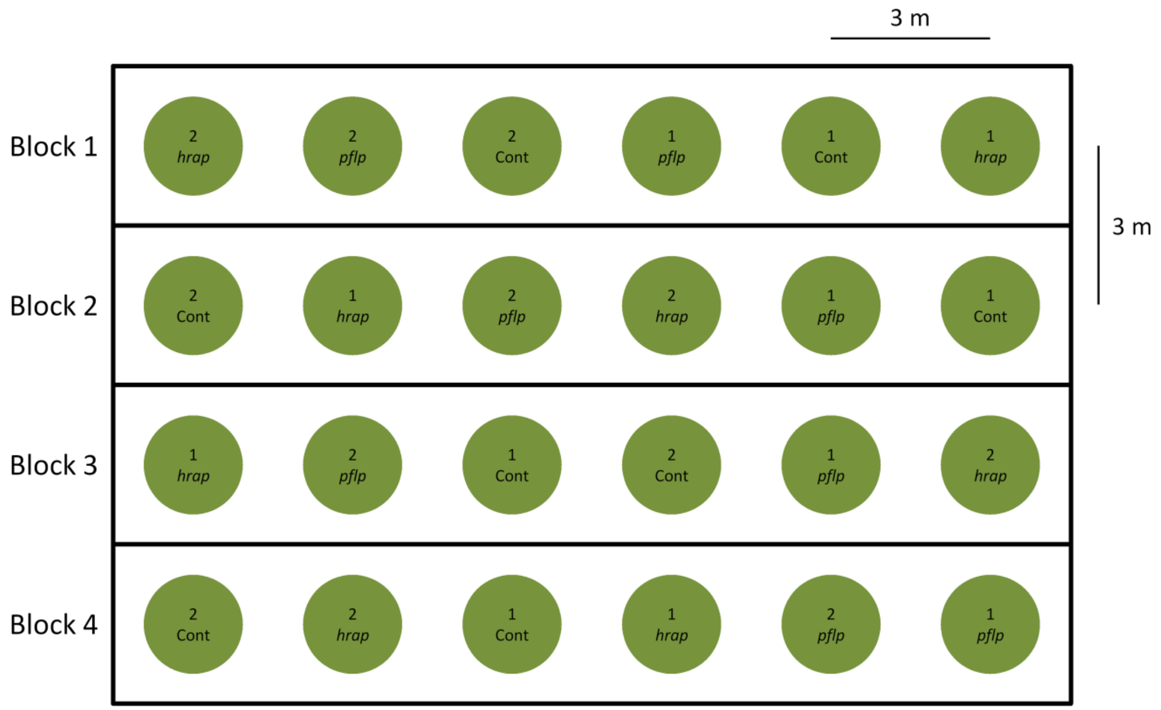

Figure S1. Randomized complete block design (RCBD) of the confined field experiment with transgenic bananas at the National Agricultural Research Laboratories (NARL) in Uganda. The cultivar under study was the East African Sukari Ndizi (AAB genome) with two different breeding lines (1 and 2), each expressing vector-inserted *hrap* (hypersensitive response assisting protein) and *pflp* (plant ferredoxin-like protein) genes. The experimental plot was surrounded by guard row plants (not depicted in this scheme).

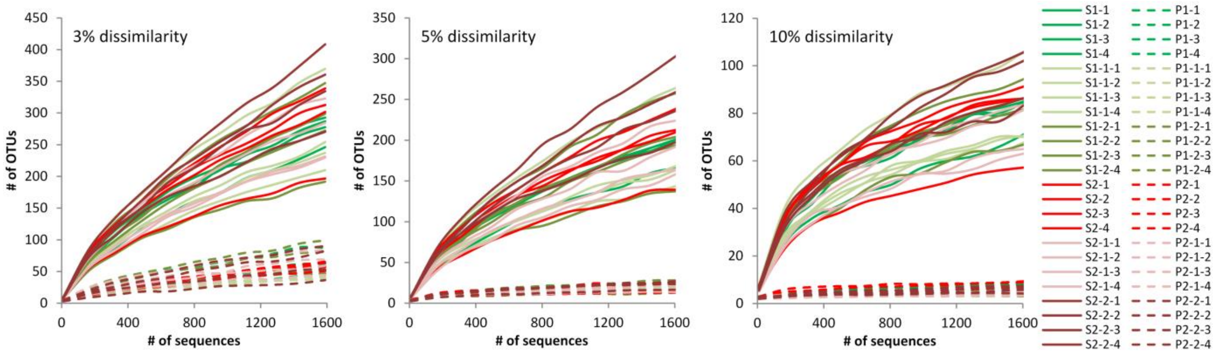

Figure S2. Rarefaction analysis for *Gammaproteobacteria*-specific 16S rRNA gene sequence libraries. Sample abbreviations indicate: (1) microenvironment (S = rhizosphere soil, P = pseudostem), (2) breeding line (1, 2), (3) genetic modification, if any (1 = *hrap*, 2 = *pflp*), and (4) independent replicate sample (1-4). For each breeding line, curves are presented from four independent replicate samples per genotype. OTUs are shown at genetic distance levels of 3%, 5%, and 10%, corresponding to the taxonomic levels of species, genera, and families.
